# Spatial memory deficits in Alzheimer’s disease and their connection to cognitive maps’ formation by place cells and grid cells

**DOI:** 10.3389/fnbeh.2022.1082158

**Published:** 2023-01-12

**Authors:** Azul Silva, María Cecilia Martínez

**Affiliations:** ^1^Facultad de Ciencias Médicas, Universidad de Buenos Aires, Buenos Aires, Argentina; ^2^Consejo Nacional de Investigaciones Científicas y Técnicas, Instituto de Fisiología y Biofísica “Dr. Bernardo Houssay”- CONICET (IFIBIO), Universidad de Buenos Aires, Buenos Aires, Argentina; ^3^Facultad de Ciencias Exactas y Naturales, Departamento de Biología Molecular y Celular “Dr. Héctor Maldonado”, Universidad de Buenos Aires, Buenos Aires, Argentina

**Keywords:** Alzheimer’s disease, cognitive map, navigation, hippocampus, entorhinal cortex, remapping

## Abstract

Whenever we navigate through different contexts, we build a cognitive map: an internal representation of the territory. Spatial navigation is a complex skill that involves multiple types of information processing and integration. Place cells and grid cells, collectively with other hippocampal and medial entorhinal cortex neurons (MEC), form a neural network whose activity is critical for the representation of self-position and orientation along with spatial memory retrieval. Furthermore, this activity generates new representations adapting to changes in the environment. Though there is a normal decline in spatial memory related to aging, this is dramatically increased in pathological conditions such as Alzheimer’s disease (AD). AD is a multi-factorial neurodegenerative disorder affecting mainly the hippocampus-entorhinal cortex (HP-EC) circuit. Consequently, the initial stages of the disease have disorientation and wandering behavior as two of its hallmarks. Recent electrophysiological studies have linked spatial memory deficits to difficulties in spatial information encoding. Here we will discuss map impairment and remapping disruption in the HP-EC network, as a possible circuit mechanism involved in the spatial memory and navigation deficits observed in AD, pointing out the benefits of virtual reality as a tool for early diagnosis and rehabilitation.

## Introduction

The passing of time affects the body, and it also has effects on the brain, these being highly variable among individuals. In particular, memory loss becomes more frequent with aging but this will not translate into dramatic changes in the life quality of most people. However, a part of the population suffers typical age-related cognitive deficits and others might experience pathological impairments such as mild cognitive impairment, or Alzheimer’s disease (AD) (Grundman et al., 2004; Hort et al., 2007; Wilson et al., 2011; Boccia et al., 2016). AD is a multi-factorial disease characterized by the progressive deterioration of many cognitive skills (Wilson et al., 2011). Though its onset and development might differ between individuals, it has three associated abnormalities: brain volume reduction (Du et al., 2007), pathological accumulation of amyloid plaques (Zhang et al., 2022), and hyperphosphorylated tau protein neurofibrillary tangles (Armstrong, 2009).

In AD patients, there is amyloid-β (Aβ) plaque deposition derived from the amyloid precursor protein (APP), which may progressively lead to tau pathology and the deterioration of brain circuit functions and cellular degeneration over several years (Sasaguri et al., 2017). The accumulation of Aβ generates high-toxicity Aβ soluble oligomers, which then aggregate into plaques of fibrillar amyloid (Drummond and Wisniewski, 2017).

Besides, in AD and the aging brain, tau proteins are hyperphosphorylated and abnormally folded. In normal physiological conditions, tau proteins stabilize the microtubules in the axon, contributing to axonal transport, development, and growth (Barbier et al., 2019). The loss of tau function is coupled with increased aggregation properties (Avila et al., 2004; DeTure and Dickson, 2019) and abnormal tau is linked to the spreading of tau pathology throughout the brain in AD (DeTure and Dickson, 2019).

The clinical diagnosis of AD is based on an observable cognitive decline and complementary PET scans which correlate with amyloid plaque deposition. While amyloid plaque deposition is one of the characteristics that define this pathology, there is a proportion of the elder population (from 5% in the 50–60 years old group up to 50% in the 70–80 years old group) with elevated plaque levels that show no cognitive impairment. For this reason, it is fundamental that the PET scan is interpreted in an adequate clinical context (Marcus et al., 2014). Finally, the definite confirmation of this pathology requires the post-mortem assessment of both amyloid plaques and tau neurofibrillary tangles (Drummond and Wisniewski, 2017).

To assess cognitive decline in AD, common clinical evaluations include tests of episodic memory, executive function, and general orientation and cognition (Rajan et al., 2015). Learning and memory are central topics in cognitive aging and AD research, yet fewer studies have investigated changes in spatial navigation (Lester et al., 2017). Remarkably, spatial disorientation is one of the first landmarks that lead to AD diagnosis. In fact, more than 60% of AD patients suffer from spatial memory deficits and wandering behaviors (Hope et al., 1994).

Here we will discuss evidence from transgenic rodent AD models with altered firing properties of hippocampal and entorhinal neurons that lead to disrupted cognitive maps and, in consequence, spatial memory deficits. Besides, we will introduce findings derived from the use of virtual reality in humans highlighting its potential use as a tool to enable an earlier diagnosis of AD or for rehabilitation.

## Cells involved in cognitive map formation

The ability to navigate an environment depends on the processing of several modalities of information. It requires the interpretation of multi-sensorial inputs and the generation of an internal representation of the space based on the animal’s prior knowledge. This was called “cognitive map” and it was first introduced by Tolman (1940). Tolman’s work had an influential value when single neuron activity with map-like representations was discovered in the hippocampal-entorhinal circuit (HP-EC) (Moser et al., 2015). The HP-EC contains a variety of neurons encoding different aspects of space crucial for its representation and for navigating through it. Hippocampal place cells are one of the best-known of all of them, having a preferential firing in precise coordinates of space (O’Keefe and Nadel, 1978). Grid cells of the MEC fire at several coordinates following a regular grid of triangles covering the entire surface of an environment (Hafting et al., 2005). This grid pattern of activity supports the representation of the distance between spatial locations, providing a spatial metric for the cognitive map. There is a vast diversity of other functional cell types in the HP-EC with a map-like organization: band cells (Krupic et al., 2012), head direction cells (Taube et al., 1990), border cells (Solstad et al., 2008; Lever et al., 2009), object cells (Høydal et al., 2019), speed cells (Kropff et al., 2015; Hinman et al., 2016), goal cells (Hok et al., 2007), reward cells (Gauthier and Tank, 2018), and even cells that encode conspecific location on the map (Danjo et al., 2018; Omer et al., 2018). The existence of all these functional types of neurons with map-like computations supports the idea that the HP-EC circuit could form the cognitive map necessary for navigation through space and daily experiences.

Under some conditions, associative memory networks need to decorrelate overlapping inputs before storing them. In response to different contexts, place cells and grid cells can change their activity, a process called remapping (Leutgeb et al., 2005). By remapping, they create specific representations for different environments. When animals explore markedly different contexts, place cells and grid cells undergo a complete reorganization of their activity (global remapping) (Muller and Kubie, 1987; Hafting et al., 2005). In response to subtle changes, place cells maintain their place code but alter their firing rate (rate remapping) (Leutgeb et al., 2005). In the HP-EC circuit, remapping could be the process that enables the neuronal pattern separation of overlapping events (Colgin et al., 2008).

For the encoding of the environment, grid cells and place cells need a reference to external landmarks. This association is likely formed during the initial visit to the territory. If the environment is modified, the grid cells reorganize their fields to encode the new dimensions (Wernle et al., 2018). Hippocampal place cells can flexibly encode changes in the environment (e.g., the addition of barriers or spatial compartmentalization), however, they do not remap after changes in the connectivity between compartments (Duvelle et al., 2021).

## Place cells and grid cells in humans

Place cells were also described in humans, with electrode recordings of the frontal and medial temporal lobes of patients while they performed a virtual-navigation task. These cells displayed responses to specific locations and views of different landmarks associated with goals within a task (Ekstrom et al., 2003). Path cells were described in electrode recordings of the EC of presurgical epilepsy patients (Jacobs et al., 2010). Moreover, grid-like activity was observed in fMRI recordings of the EC of patients performing a virtual reality (VR) navigation task analog to rodents’ foraging tasks (Doeller et al., 2010). Not only this hexagonally distributed fMRI signal is present when subjects actively navigate a virtual environment but also when they imagine moving through it (Horner et al., 2016).

In humans, grid-like representations that support spatial memory were found in the HP-EC system, which is associated with autobiographical memory and imagery (Doeller et al., 2010; Bellmund et al., 2016; Horner et al., 2016) and in the EC and medial prefrontal cortex in the processing of odor landscape (Bao et al., 2019). Grid-like activity was also found in goal-directed tasks not related to spatial navigation, where abstract non-spatial relationships were coded into maps resembling the ones used for spatial navigation (Garvert et al., 2017). Given that a cognitive map is a way of organizing information to build an internal representation of the environment, it is highly likely that grid coding underlies other cognitive processes besides topographical representations (Ekstrom et al., 2020; Peer et al., 2021).

In sum, the neurons in the HP-EC circuit can encode multiple aspects of an experience besides the position in space, suggesting that they have a crucial role not only in spatial navigation but also in episodic memories. Further, they exhibit pattern separation/completion activity that allows spatial disambiguation between similar memories, which in turn enables the successful retrieval of mnemonic traces (Kyle et al., 2015). Hence, they are a unique target to study spatial memory impairment and wandering behavior that AD patients suffer.

## Transgenic models of AD and space coding impairment

The histopathological post-mortem analysis of AD patients shows that amyloid plaques and tau pathology are present together, however, these are less commonly found in AD models because of the technical limitations to obtaining consistent and abundant expression of both plaques and tangles (Drummond and Wisniewski, 2017). No animal models can fully recapitulate AD, nevertheless, they allow a better understanding of the mechanisms that underlie navigation impairment, disorientation, and wandering. Recent findings in transgenic mice with amyloid plaques or neurofibrillary tangles in the HP-EC circuit show deficits in place-cell and grid-cell activity, leading to altered cognitive maps and difficulties in solving spatial tasks, pointing to these neurons as key players in the navigational and memory decline observed in AD (For summary, see Table 1).

**TABLE 1:** List of the AD models discussed.

Model	Sex	Age (months)	Amyloid plaques	Neurofibrillary tangles	Neurode- generation	Behavioral test	HP activity affected	EC activity affected	References
rTg4510	Male and female	7–9	NA	Yes	Yes	Free context exploration in linear tracks and open field	Impaired place cells spatial coding PCs present repetitive firing sequences among environments	NA	Cheng and Ji, 2013
rTg4510	Male	7–8	NA	Yes	Yes	Morris Water Maze Linear track	Impaired place cells spatial coding Altered network oscillations Sharp wave ripples	NA	Booth et al., 2016
rTg4510	Male	6–7	NA	NA	NA	Linear track	NA	Impaired grid cells spatial coding Altered theta and gamma power during locomotion	Ridler et al., 2020
3xTg	Male	8–9	Not at 8–9 months but present at 11–12 months.	NA	Yes	Circular track	Impaired spatial coding Altered theta and gamma rhythms	NA	Mably et al., 2017
APP–PS1	Male and female	6	Yes	NA	NA	Spatial and non-spatial rewarded task	Inflexible task and space representations. Altered population activity.	NA	Cayzac et al., 2015
J20 APP	Male and female	3 and 7	Yes	NA	Yes	Free context exploration in open field Path integration task	Place cells preserved space coding activity	Grid cells with reduced spatial periodicity. Non-spatial cells remained intact.	Ying et al., 2022
APP KI	Male and female	3–5 and 7–13	Yes	NA	NA	Context discrimination task Free context exploration in open field Remapping task	Yes. Impaired remapping.	Yes, early onset. Severe impairment in spatial tuning.	Jun et al., 2020
Tg2576	Male	3 and 16	Yes	NA	NA	Forced-choice spatial alternation T-maze task	Impaired PC spatial coding in aged (16 months) but not young (3 months) transgenic mice	NA	Cacucci et al., 2008
EC-TAU	Male and female	14 and +30	NA	Yes	Yes	Morris Water Maze T-maze task	NA	Impaired GC spatial coding and enhanced theta rhythm in 30+ months mice	Fu et al., 2017
fAD	Male	8–14	Yes	NA	NA	Tactile morph maze	Pattern separation deficit in DG Pattern completion deficit in CA3	NA	Rechnitz et al., 2021
TgF344 rats	Female	5–12	Yes	Yes	Yes	Object and object-in-location recognition memory tasks Free exploration in circular track	Reduced spatial fidelity in CA2 and CA3	NA	Galloway et al., 2018
TgF344 rats	Female	6, 9, 12	Yes	Yes	Yes	Morris Water Maze, Radial Arm Water Maze	Western blots show increased Aβ. Impaired place coding in CA2 and CA3	Western blots show increased Aβ	Bernaud et al., 2022

NA, Not Assessed.

Place cell abnormalities have been reported in different mouse models of AD. rTg4510 mice (7–8 months) with advanced tau pathology and progressive neurodegeneration showed disrupted place cell coding. Although place cells did not show place specificity, they displayed robust firing sequences. However, these sequences were similar among different environments, suggesting that they lost their ability to represent the animal’s current surroundings (Cheng and Ji, 2013). Further work with this model showed spatial memory deficits accompanied by alterations in the intrinsic properties of CA1 pyramidal neurons leading to aberrant CA1 network oscillations and altered place cell firing properties (Booth et al., 2016).

Disturbed CA1 local oscillatory activity and impaired place coding were also described in the APP-PS1 model. Six months APP-PS1 mice have increased amounts of soluble and insoluble Aβ and show degraded spatial information processing and inflexible representations in their place cells during reversal learning in an operant conditioning task (Cayzac et al., 2015). At 11 months Aβ-expressing APP mice did not improve their place cell coding through learning, producing spatial codes of lower resolution that correlate with delayed learning (Zhao et al., 2014). Similarly, place cells with a degraded neuronal representation of the environment were also reported in aged – but not in young – Tg2576 mice and were associated with deficits in spatial memory tasks (Cacucci et al., 2008). In accordance with these findings, 3xTg mice develop a progressive accumulation of plaques and tangles, and show disrupted coordination of place cell firing by hippocampal theta and gamma rhythms (Mably et al., 2017). Theta and gamma rhythms are involved in many hippocampal cognitive processes, such as encoding and retrieval of episodic memories (Vertes et al., 2004; Nyhus and Curran, 2010). Network dysfunctions have been proposed to underlie some of the fluctuations in neurological functions observed in AD (Palop et al., 2006). Accordingly, theta and gamma dysfunctions were observed in several AD models, being reduced gamma and theta power a common feature among them (Villette et al., 2010; Rubio et al., 2012; Ittner et al., 2014; Schneider et al., 2014; Gillespie et al., 2016; Iaccarino et al., 2016).

Unimpaired grid coding in the HP-EC circuit is necessary for path integration in spatial navigation. The ablation of NMDA glutamate receptors in the retro-hippocampal region reduced spatial selectivity and grid spatial periodicity of the MEC principal cells (Gil et al., 2018). Likewise, mice lacking GluA1-containing AMPA receptors of the MEC showed difficulties in a homing task when allocentric cues were reduced (Allen et al., 2014). Furthermore, in transgenic models of tau pathology, excitatory neuronal loss and grid cell dysfunction in the MEC were associated with poor spatial coding. In 30-month-old EC-tau transgenic mice, accumulated tangles altered the network properties: grid cell firing rate was reduced, grid fields were destabilized, spatial information content was diminished and theta power was enhanced. At the stage where these alterations appeared, mice showed deficits in spatial learning and memory tasks, such as Morris Water Maze and T-maze test (Fu et al., 2017). Remarkably, the disturbances were observed in dorsal MEC, following previous work that showed that dorsal MEC, and not ventral, is preferentially vulnerable to pathological tau (Booth et al., 2016).

Grid coding was impaired in APP-KI and J20 APP mice lines expressing Aβ protein in the hippocampus and the MEC. Spatial memory deficits are observed in >6 months male and female mice and, though they present a mild deterioration in their place cells’ activity, the most adverse effect is observed in the grid cells of the MEC and it has an earlier onset (Jun et al., 2020; Ying et al., 2022). In particular, APP-KI mice had impairments in remapping that emerged first in grid cells and then in place cells. As with the amyloid models, in the tauopathy model, rTg4510 mice lost grid cell periodicity and, as a result, exhibited impaired path integration. Hippocampal place cell coding was less affected than the grid cell coding of the MEC (Ridler et al., 2020).

Remapping deficits were also observed in a mice model of familial AD (APPSwe + PSEN1/ΔE9). Pattern separation and pattern completion were affected in a regional way, where pattern separation was reduced in the dentate gyrus, and pattern completion was reduced in the CA3 region (Rechnitz et al., 2021). Although it is not clear whether deficits in CA3 are a consequence of corrupted inputs (from the DG or EC) or arise as an independent regional deficit, these results suggest a network-level information processing impairment.

TgF344 female rats with increased gliosis, amyloid plaques, and neurofibrillary tangles showed working and reference memory impairments in the radial-arm maze and Morris Water maze following a linear development (6, 9, and 12 months). Remarkably, the 9 months group conserved its working memory and had a distinctive correlation between physiological and brain outcomes, putting forward that critical processes might be taking place at this intermediate point in the course of the disease (Bernaud et al., 2022). Another work with this model showed that the behavioral deficits observed at 12 months were accompanied by disrupted place cell coding (Galloway et al., 2018), in consonance with evidence in mice models.

Together, these results show that place cell and grid cell perturbations are a common feature of the vast majority of AD models and, in most cases, they are accompanied by a decline in spatial navigation and memory performance. Interestingly, in mice models where both place and grid aberrant activity was reported, the most adverse effects were found in grid cells. Moreover, the effect on grid cells had an earlier onset, resembling what has been described in patients (Braak and Braak, 1991). Overall, evidence from transgenic models suggests that hippocampal place cells can form place fields without entorhinal cortex input in these AD models. Thus, deficient place representation could first arise from the lack of the allocentric reference provided by grid coding and, as the pathology progresses, place cell coding becomes disrupted too. In humans, the deterioration of EC activity could be at the basis of spatial disorientation and wandering.

## Virtual reality as a tool to assess AD

The use of virtual navigation tasks alongside fMRI enabled the discovery of grid-like activity in humans (Ekstrom et al., 2003). VR correlates highly with real-world navigation (Puthusseryppady et al., 2020) and allows the representation of large areas into a smaller space. Though that VR lacks vestibular and proprioceptive activity, visual, planning, and memory systems are engaged in these types of tasks (Epstein et al., 2017).

Younger and older adults show different navigational skills in a VR homing task, which could be driven by increased noise in spatial representations in the HP-EC network (Bates and Wolbers, 2014). Using fMRI and VR, Kunz et al. (2015) studied neural correlates of spatial navigation in the EC in control study participants and individuals at risk of developing AD. This group showed reduced grid-cell–like activity and altered navigation many decades before the onset of the disease. Moreover, in an immersive VR Object-Location test, patients at risk of developing AD had poorer performance than their age-matching controls, making it a potential tool for the diagnosis of the development of the disease (Castegnaro et al., 2022). Accordingly, AD patients showed impairments in VR tasks designed to study navigational skills, however, their performance could not be used to predict the disorientation degree they may have in the real world (Coughlan et al., 2020).

Virtual reality offers a controlled secure environment with multimodal stimulation that can be standardized and used for navigation and memory assessment in elder patients. As it allows repetitive practice, feedback on the performance, and the incorporation of different stimuli VR also opens up the possibility of generating rehabilitating tools to improve spatial memories and navigational skills (Serino and Riva, 2013; Colombo et al., 2017). Thus, VR represents a unique tool that could be used for early diagnosis and rehabilitation as well.

## Conclusion

The earliest stages of AD are characterized by the formation of mature tangles in the EC and are accompanied by disorientation and confusion when navigating familiar places. Several mechanisms contribute to the deterioration of the HP-MEC circuit in AD. In pathological conditions, excessive amyloid plaque deposition and neurofibrillary tangles affect neuronal metabolism, axonal transport, and growth, and increase neurotoxicity and inflammation, among other phenomena (Domínguez-Álvaro et al., 2021; Kobro-Flatmoen et al., 2021). Though AD models may not reproduce the pathological development in its entirety, they bring extremely valuable information on the evolution of the disease. For instance, both clinical evidence and transgenic models show that damage to the grid cells in the MEC is detrimental to navigation and it starts earlier than in the hippocampal place cells (Jun et al., 2020; Richter et al., 2020; Ying et al., 2022). Remarkably, MEC’s neurons are among the first to exhibit degeneration as shown by anatomical studies in AD patients (Van Hoesen et al., 1991; Gómez-Isla et al., 1996).

Grid coding has a key role in cognitive map formation. Cognitive maps are representational systems that allow navigating the environment but also physical, social, and conceptual spaces. Given all these characteristics, the impairment in cognitive maps has multiple profound effects on the lives of AD patients. The development of biomarkers (Holbrook et al., 2020; Richter et al., 2020; Matuskova et al., 2021; Billette et al., 2022) together with cognitive (Vlcek and Laczo, 2014) and VR tests (Bates and Wolbers, 2014; Coughlan et al., 2018, 2020; Castegnaro et al., 2022) that can detect early changes in the MEC could be crucial for the prevention and early diagnosis of AD. Considering spatial navigation is less attached to verbal, cultural, and educational biases than other tests (Coughlan et al., 2018) and the possibility to translate VR to different locations, there is a unique window of opportunity toward a global approach in the diagnostic and treatment of AD.

## Author contributions

Both authors listed have made a substantial, direct, and intellectual contribution to the work, and approved it for publication.
